# Three-Dimensional Planimetry Assessment of Dental Plaque-Covered Area Reduction after Rinsing with 0.2% Sodium Hypochlorite Solution as Part of a Guided Biofilm Therapy^®^ Protocol—Pilot Longitudinal Study

**DOI:** 10.3390/biomedicines12102326

**Published:** 2024-10-12

**Authors:** Georgios Kardaras, Marius Boariu, Vadym Varlamov, Claudiu Vintila, Simina Boia, Alla Belova, Darian Rusu, Monika Machoy, Sorina Mihaela Solomon, Stefan-Ioan Stratul

**Affiliations:** 1 Department of Periodontology, Faculty of Dental Medicine, Anton Sculean Research Center for Periodontal and Peri-Implant Diseases, “Victor Babes” University of Medicine and Pharmacy, 300041 Timisoara, Romania; kardaras.georgios@umft.ro (G.K.); vadimvarlamov19@gmail.com (V.V.); simina.boia@umft.ro (S.B.); alla.belova@umft.ro (A.B.); rusu.darian@umft.ro (D.R.); stratul.stefan@umft.ro (S.-I.S.); 2 Department of Endodontics, Faculty of Dental Medicine, TADERP Research Center, “Victor Babes” University of Medicine and Pharmacy, 300041 Timisoara, Romania; 3 Private Practice, 300176 Timisoara, Romania; claudiuvintila@ymail.com; 4 Department of Periodontology, Pomeranian Medical University, 70-204 Szczecin, Poland; m.machoy@gmail.com; 5 Department of Periodontology, Faculty of Dental Medicine, Gr.T.Popa University of Medicine and Pharmacy, 700115 Iasi, Romania; drsolomonro@gmail.com

**Keywords:** Guided Biofilm Therapy^®^, NaOCl rinsing solution, 3D intraoral scanning

## Abstract

**Background/Objectives**: Less often employed as a rinsing solution for controlling oral biofilms, NaOCL was used in oral rinses at various concentrations in steps 1 and 4 of periodontal therapy. The aim of this study was to quantitatively evaluate the biofilm-disruptive properties of a 0.2% NaOCl solution in standardized oral rinses using dedicated plaque-disclosing agents and 3D scanning methods in patients undergoing the regular Guided Biofilm Therapy^®^ protocol. **Methods**: Eight patients with at least 20 teeth present evenly distributed between the two arches were included. After 24 h of refraining from oral hygiene, dental arches were stained with a disclosing agent, the subjects rinsed for 20 s, clinical photographs and 3D scans were performed, subjects rinsed again for 20 s, photographs and 3D scans were performed again, and then the GBT^®^ protocol was resumed as usual. Data representing areas covered with dental plaque were acquired using the “Medit Scan for Clinics” software and then underwent a post-processing and rendering process. The outcome variable was the percent reduction in the plaque-covered areas. **Results**: For the upper jaw, the estimated mean percent reduction in the biofilm-covered area was 39.65%, while for the mandible, it was 38.26%. The analysis of individual photographs revealed changes in the plaque-covered areas and reductions in the color intensity of the residual plaque-covered areas under identical lighting conditions. **Conclusions**: When analyzed using 3D intraoral scanning, the 0.2% NaOCl rinsing solution seems to be a clinically efficient disruptor/dissolvent of the oral biofilm, both when integrated into modern protocols of periodontal therapy like GBT^®^ and for home self-care.

## 1. Introduction

The control of oral biofilm, ideally achieved through a combination of mechanical and chemical therapeutical approaches, is essential for the prevention of periodontitis—a bacteria-driven disease. Over the last two decades, the performance of various antimicrobial formulations has been reported in several systematic reviews [1,2], mostly focusing on one specific agent or a limited group of agents. Over time, different types of agents have been used in oral rinses, the most common being the chlorhexidine (CHX) [3,4], cetylpyridinium chloride (CPC) [5,6], triclosan zinc citrate [7], and essential oils (Eos) [8].

The main purpose of an antimicrobial oral rinse formulation is to create “dysbiosis” in the structure of biofilm, in order to prevent its formation and stability. In this direction, sodium hypochlorite (NaOCl) stands out for its ability to dissolve most organic matter during endodontic treatment [9]. NaOCl has also been used in oral rinses with concentrations varying from 0.05 to 0.25% rinsing solution in step 1 of periodontal therapy [10,11,12,13].

Recently, Guided Biofilm Therapy was used as a systematic, predictable solution for dental biofilm management in professional prophylaxis using state-of-the-art AIRFLOW^®^, PERIOFLOW^®^ and PIEZON^®^ technologies. It consists of treatment protocols based on individual patient diagnosis and risk assessment in order to achieve optimal results. The treatment minimizes the use of power and hand instrumentation and follows the European Federation of Periodontology (EFP) recommendations on Professional Mechanical Plaque Removal (PMPR) and Oral Hygiene Instructions (OHI) for Home Care of the European Federation of Periodontology (EFP) [14].

However, properly and precisely detecting the oral biofilm to be effectively removed is more than an intangible challenge, as biofilm is generally colorless. Several biofilm identification methods have been used over time, the most frequent being the disclosing method.

Plaque is visible with the naked eye, especially in larger amounts; however, disclosing it is a more efficient way to demonstrate its presence and persistence to a patient at the chair side. For many therapeutic reasons, clinicians need to be able to assess plaque levels. This can be achieved using disclosing substances that make plaque visible, helping the patients and dental professionals to assess its quantity and location [15].

Researchers can use this combined with index scales and charting of the plaque areas to make comparisons between treatment visits and validate control and experimental therapies, as well as the efficiency of oral hygiene products [16].

Oral biofilms can be properly identified with special staining agents such as iodine, gentian violet, or erythrosine alone or combined with a blue dye, basic fuchsin, fast green, food dyes, fluorescein, or two-tone disclosing agents in the form of tablets, solutions, wafers or mouth rinses [17,18]. Other methods use erythrosine combined with a blue light [19], combinations of fluorescent agents and laser techniques [15,16]. After application, these agents color the areas of the oral cavity where biofilm is present, and depending on the age of the plaque, they generate a specific coloration intensity [20]. Among properties of plaque-disclosing agents, persistence—meaning that the color should not rinse off with ordinary rinsing methods, air–water spraying, or salivary flow—is one of the most important [21].

Dental plaque has the ability to retain a large number of dye substances, a property that is related to the polarity difference between the components of the plaque and the colorants. The particles are attracted to the surface by electrostatic interaction between proteins and polysaccharides through hydrogen bonds [22]. Disclosing solutions can be applied using several methods including direct application (by swab or a small cotton pellet), rinsing, tablets or wafers (by chewing), and dentifrices (the plaque-disclosing agents are incorporated in dentifrices, and they disappear after proper brushing) [23].

In recent years, some of these identification techniques have enabled advanced plaque analysis techniques like “digital plaque image analysis” [15] and 3D intra-oral scanning methods (“planimetric plaque analysis”) [24,25,26,27,28,29]. Intraoral scanning with 3D technology is a suitable tool for whole-mouth plaque assessment and monitoring [27,28,30,31]. This method allowed for better visualization and quantification of areas covered with oral biofilms on all aspects of dental arches that can be scanned.

However, to date, changes in plaque presence before and after antimicrobial procedures have not been evaluated using advanced evaluation techniques; most available research has focused on the suitability of these techniques for valid planimetric measurements and plaque monitoring of the technique (e.g., 3D intraoral scanning vs. intraoral imaging, before and after tooth brushing) and rather than evaluating the efficacy of specific plaque removal methods [27,28]. More recently, the capacity for detecting dental plaque was tested using 3D intraoral scans in comparison with clinical findings [29] or among various brands of scanners [32].

Furthermore, there are currently no hypotheses explaining the detailed mechanism by which biofilm-disrupting antimicrobials reduce the amount of plaque, and various disclosure-based methods are expected to help elucidate these changes. Even less explained and measured is the cumulated reductive effect of combinations of mechanical disruption and chemical dissolution/disruption of biofilm on dental crowns.

The aim of this pilot prospective study was to quantitatively evaluate the biofilm-disruptive properties of a 0.2% NaOCl solution in standardized oral rinses by using dedicated plaque-disclosing agents and advanced 3D scanning methods in patients undergoing the regular Guided Biofilm Therapy^®^ protocol. The nature of biofilm disruption under the action of NaOCl solution was also hypothesized.

## 2. Materials and Methods

Between February 2023 and February 2024, this pilot prospective study enrolled eight subjects—outpatients at the Clinic of Periodontology of the Faculty of Dental Medicine of the “Victor Babeş” University of Medicine and Pharmacy Timișoara, Romania. The study protocol was approved by the Research Ethics Committee of the ‘Victor Babeş’ University of Medicine and Pharmacy (approval No. 56/2020).

The study was conducted over a period of 12 months (February 2023–February 2024), and the procedures followed were in accordance with the Declaration of Helsinki, the ethical standards of the responsible committee on human experimentation and rules of good practice in biomedical and educational research. All patients were informed about the nature and purpose of the study, and each of them signed an informed consent document granting permission for the dental procedures.

### 2.1. Study Population

Assuming that the true mean percent reduction is 40% with the corresponding SD of 20%, with 8 subjects, we have 80% power to conclude that the mean percent reduction is different from 15% at a significance level of 0.05.

The study population consisted of 8 patients, 4 males and 4 females (mean age 48 years; range 26–70 years).

Inclusion criteria:

- Patients systemically healthy with at least 20 teeth present (natural or artificial crowns), evenly distributed between the two arches;
- Patients willing to participate and who signed the informed consent;
- Patients not having practiced oral hygiene for 24 h before the procedure.

Exclusion criteria:

- Patients with known intolerances, allergies, and hypersensitivity to the components of the plaque-disclosing agent (glycine, etil paraben, Cetilpiridiniumclorid, Povidone Iodine, Erytrosine, blue dye) and NaOCl.

### 2.2. Clinical Procedures

The standard GBT^®^ eight-step protocol normally includes [14]: (1) assessment and infection control; (2) biofilm disclosure in problematic areas using EMS Biofilm Disclosure (E.M.S. Electro Medical Systems S.A., Nyon, Switzerland); (3) based on the appearance of biofilm, the patient is motivated to emphasize the importance of oral hygiene and prevention; (4) the handpiece Airflow^®^ Max (E.M.S. Electro Medical Systems S.A., Nyon, Switzerland) is used for biofilm, stains, and early calculus removal from natural teeth, restorations, and implants; (5) the handpiece Perioflow^®^ (E.M.S. Electro Medical Systems S.A., Nyon, Switzerland) (and, wherever suitable, the new and slimmer Perioflow^®^ nozzle) is used for biofilm removal in periodontal pockets ≥ 4 mm, with Airflow^®^PLUS (E.M.S. Electro Medical Systems S.A., Nyon, Switzerland) powder; (6) removal of remaining calculus with minimally invasive EMSPiezon^®^ PS (E.M.S. Electro Medical Systems S.A., Nyon, Switzerland) or with Piezon^®^P1 MAX (E.M.S. Electro Medical Systems S.A., Nyon, Switzerland); (7) final check for remaining biofilm; and (8) recall scheduled according to the risk assessment.

**Figure 1:**
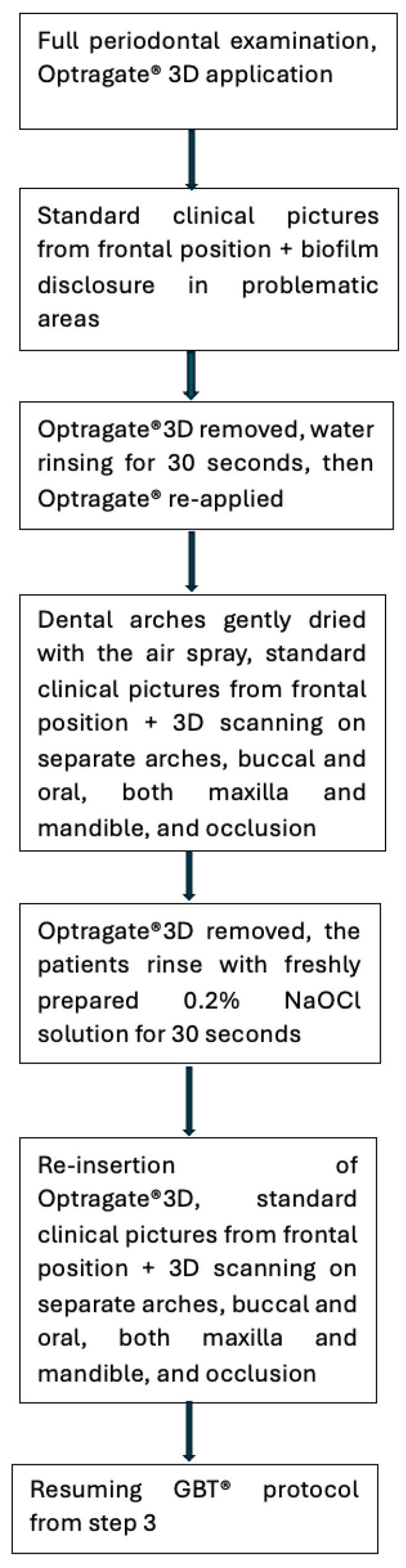
Study flowchart.

**Figure 2:**
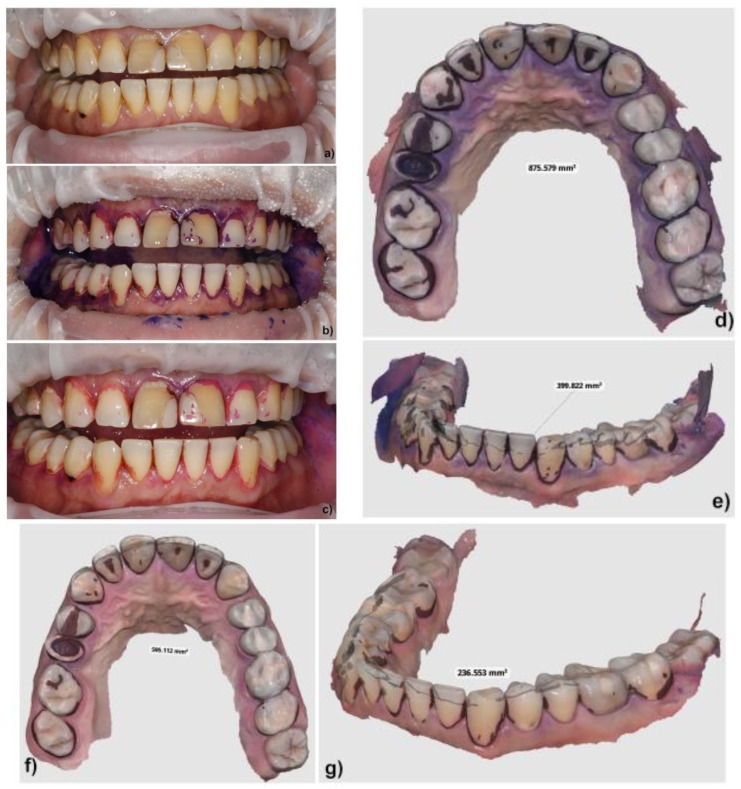
Case 1. (**a**) clinical image before staining; (**b**) photograph of residual plaque after 30 s of rinsing with water; (**c**) photograph of residual plaque after rinsing with 0.2%NaOCl; (**d**,**e**) 3D scan of the residual plaque after 30 s of rinsing with water and the measured value of plaque-covered areas of the maxilla and mandible; (**f**,**g**) 3D scan of residual plaque after 30 s of rinsing with 0.2%NaOCl and the measured value of plaque-covered areas of the maxilla and mandible.

**Figure 3:**
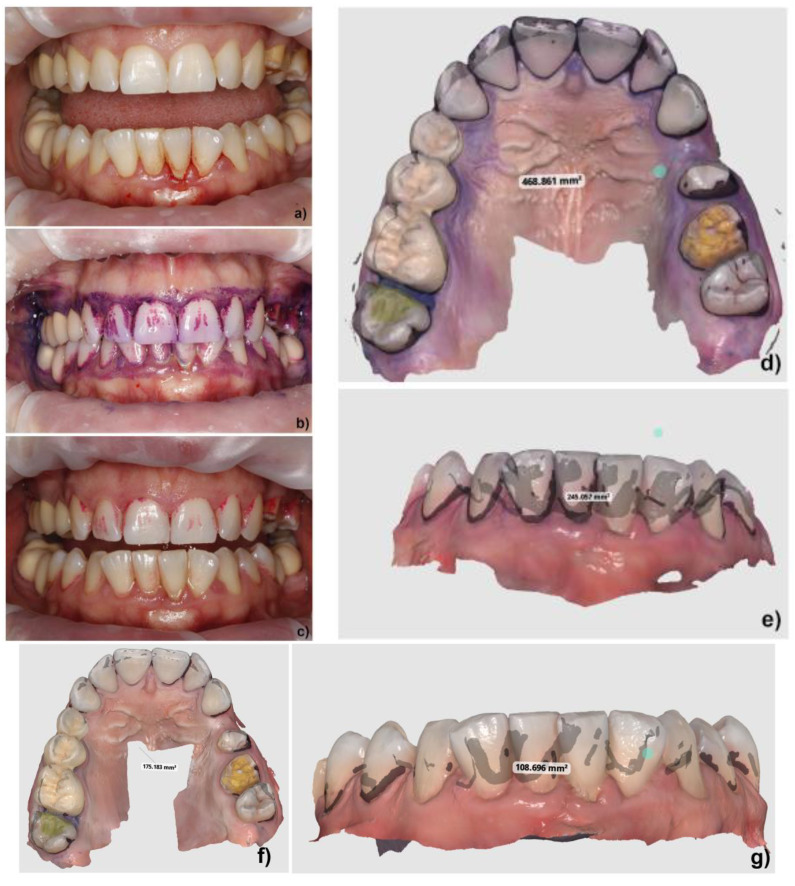
Case 2. (**a**) clinical image before staining; (**b**) photograph of residual plaque after 30 s of rinsing with water; (**c**) photograph of residual plaque after rinsing with 0.2%NaOCl; (**d**,**e**) 3D scan of the residual plaque after 30 s of rinsing with water and the measured value of plaque-covered areas of the maxilla and mandible; (**f**,**g**) 3D scan of residual plaque after 30 s of rinsing with 0.2%NaOCl and the measured value of plaque-covered areas of the maxilla and mandible.

In our study, the protocol was personalized as follows: the treatment was initiated with a full periodontal examination for each patient, including implants and peri-implant tissues, followed by the application of an Optragate 3D (Ivoclar, Vivadent, Schaan, Liechtenstein) dental, lip, and cheek retractor. Standard clinical pictures from the frontal position were taken. Biofilm disclosure was performed in problematic areas using EMS Biofilm Disclosure (pre-soaked pellets based on erythrosine and patent blue, for 2-tone biofilm disclosure: reddish for new biofilm, blue for mature biofilm) [33]. (Figure 1). The Optragate was removed, the patient rinsed with water for 30 s, and then the Optragate was re-inserted. The arches were gently dried with the air spray and then standard clinical pictures from the frontal position were taken again, followed by 3D scanning on separate arches, buccal and oral, both maxilla and mandible, and occlusion, using the Medit i700 intraoral scanner, wired version (Medit SG PTE. Ltd., Yeongdeungpo District, Seoul, Republic of Korea). The Optragate was removed again, and the patients rinsed with freshly prepared 0.2% NaOCl solution for 30 s. After re-insertion of the Optragate, standard clinical pictures from the frontal position were repeated, followed by 3D scanning on separate arches, buccal and oral, both maxilla and mandible, and occlusion, using the Medit i700 (Medit SG PTE. Ltd., Yeongdeungpo District, Seoul, Republic of Korea) intraoral scanner (Figure 2 and Figure 3). Then, the GBT protocol, as described above, was resumed from step 3.

### 2.3. Data Acquisition

Intraoral scans were captured using the i700 intraoral scanner, wired version, synced with the “Medit Scan for Clinics” data-capturing software, version 1.11.1, for Windows 11 (Medit SG PTE. Ltd., Yeongdeungpo District, Seoul, Republic of Korea). Device calibration was performed every time prior to the image-capturing protocol, in order to use the device in optimal conditions. Switching the color of the scanning light used in the data-acquiring process from blue light to white light resulted in richer color contrasts and more reliable color mapping of the textures, creating more clear margins and better demarcation between biofilm-covered areas and clean surfaces.

Data were acquired using the HD Capture Mode for increased texture resolution. Clinicians conducting the research adhered to a reproducible and precise full arch scan strategy, using well-defined continuous movements and placement of the device in relation to the areas of interest, aiming for consistent and accurate mapping of the hard and soft tissue surfaces with every scan involved in the research protocol. Any unaligned data that resulted from faulty movements during the scanning process were dismissed and recaptured until proper alignment with the rest of the dental arch was achieved, thus avoiding the need for any manual alignment or use of the “Smart Stitching” feature. The “Smart Color Filtering” feature was also disabled in order to avoid any unwanted automatic color corrections of the scanned surfaces.

After scanning and storing, the raw data underwent a post-processing and rendering process using “Medit Link” software version 3.2.1 build 206 (Medit SG PTE. Ltd., Yeongdeungpo District, Seoul, Republic of Korea), which were saved as STL files. The resulting data were imported into “Medit Design” software version 2.1.4 for further analysis and processing (Medit SG PTE. Ltd., Yeongdeungpo District, Seoul, Republic of Korea), carried out by accessing the “Measurement Mode”. This allows for area measurements on 3D data. In this specific mode, clinicians used the “Calculate Area by Selection” tool to provide highly accurate selection of dental biofilm-covered areas marked in contrasting color by the disclosing agent applied before scanning. The “Area Selector” tool allowed for the use of different brush sizes for the selection tool, thus increasing the accuracy of selecting areas of interest. Resulting area calculations were performed. To better illustrate all stained areas on a given dental arch (maxillary and mandibular) before and after rinsing, MP4 videos were also recorded.

### 2.4. Data Analysis

As the sample size in this pilot study was small, means and standard deviations were initially used to describe the data set. They were calculated for all stained areas before and after rinsing with the 0.2% NaOCl solution and for the differences (both expressed in mm^2^ and in percentages) between stained areas before and after rinsing. The values were calculated for the maxilla and mandible, cumulating the buccal, occlusal, and oral surfaces. Cumulated minimum values, cumulated maximum values, range, sum, mean, median, standard deviation and variance were analyzed using the software online CalculatorSoup® [34].

Statistical analyses were performed using SAS 9.4 (SAS Institute, Cary, NC, USA). The outcome variable was the percent reduction. This choice was based on the information provided in the literature that a percent reduction of 15% is clinically significant [35]. In order to have independent observations, as required by the usual statistical methods, we performed separate analyses for the upper and lower jaw. For each one of these two analyses, the evaluation of the normality of the percent reduction variable using the Kolmogorov–Smirnov test was performed. In the absence of violations of the normality assumption, the mean percent reduction was estimated, and the corresponding 95% confidence interval was constructed. To relate the study’s findings to the relevant literature, a one-sample two-sided *t*-test was performed to determine whether the mean percent reduction was above or below 15%.

## 3. Results

The mean total stained area of the maxilla decreased from 475.85 mm^2^ to 294.53 mm^2^, resulting in a stained area variation of 181.32 mm^2^ (39.651%) (Table 1).

The mean total stained area of the mandible decreased from 418.58 mm^2^ to 260.80 mm^2^, resulting in a stained area variation of 157.78 mm^2^ (38.26%) (Table 2).

When cumulating the results for the entire mouth, the mean total stained area decreased from 447.22 mm^2^ to 277.67 mm^2^, resulting in a stained area variation of 169.55 mm^2^ (38.95%) (Table 3).

For the upper jaw, the result of the Kolmogorov–Smirnov test did not indicate statistically significant departures from normality (*p* > 0.150). The estimated mean percent reduction was 39.65%, and the corresponding 95% confidence interval was (26.51%, 52.80%). The 15% value is not included in the 95% confidence interval, indicating that the mean percent reduction is significantly higher than 15%, as also indicated by the result of the one-sample *t*-test, which shows that the mean percent reduction is statistically significantly different from the clinically significant value of 15% (*p* = 0.003) (Table 4).

The results were similar for the lower jaw, although the 95% confidence interval was wider, and the *p* value was larger but still below the significance level of 0.05. Specifically, the result of the Kolmogorov–Smirnov test did not indicate statistically significant departures from normality (*p* > 0.150). The estimated mean percent reduction was 38.26% and the corresponding 95% confidence interval was (18.49%, 58.03%). Again, the 15% value was not included in the 95% confidence interval, indicating that the mean percent reduction is significantly higher than 15%, as also indicated by the result of the one-sample *t*-test, showing that the mean percent reduction is statistically significantly different from 15% (*p* = 0.027) (Table 5).

The analysis of individual photographs taken before and after rinsing revealed, in all patients, not only changes in the plaque-covered areas but also reductions in the color intensity of the residual plaque-covered areas from deep purple to intense pink, under identical lighting conditions.

## 4. Discussion

The aim of the present study was to evaluate the effect of a 0.2% NaOCl rinsing solution on the reduction in biofilm areas on crowns belonging to complete dental arches, in a clinical setup as part of a modified GBT protocol. An abundance of research exists on the assessment of oral plaque reduction using various therapeutical methods; however, with regard to rinsing with NaOCl solutions, the vast majority of studies report in vitro experiments [36,37,38].

As far as the authors know, this is the first study to use 3D planimetric analysis to evaluate the reduction in plaque-covered oral area following the dissolutive/disruptive action of a potent antimicrobial, e.g., natrium hypochlorite. Moreover, as far as we know, the literature contains no study of 3D-based planimetric measures addressing the reduction in biofilm-covered area using other antimicrobials employed chairside or as home self-care rinsing for anti-inflammatory and plaque-control purposes.

According to EFP S3 level Clinical Practice Guideline, the first step in the treatment of periodontitis stages I–III states is aimed at motivating the patient to undertake successful removal of supragingival dental biofilm and risk factor control. This should be implemented in all periodontitis patients [39].

Because NaOCl has excellent anti-microbial properties and is safe to use, it can be used as a mouthwash. This, combined with its low cost and availability in most households, means that patients could dilute inexpensive basic household bleach to reach a concentration that is better tolerated, e.g., 0.2% [40].

In 1984, the American Dental Association (ADA) recommended NaOCl for use as a topical antimicrobial. It can be used as a rinsing or irrigating solution by patients during steps 1 (supragingival biofilm control) and 2 (subgingival instrumentation) of periodontal therapy and during periodontal maintenance at various concentrations [10,40,41,42,43,44,45,46,47,48].

Current methods for the quantification of plaque include traditionally disclosing agents and the calculation of plaque indices; planimetric plaque methods (based on photographic images of the teeth, which can utilize an index known as the Plaque Percent Index, or PPI [18,49,50]); and novel techniques including fluorescein disclosure and digital plaque image analysis (DPIA) [15,51], plaque quantification using 3D co-ordinate data [24,25], and plaque detection using quantitative light-induced fluorescence (QLF) [51,52].

Even if planimetry has been used with many variations to analyze conventional or fluorescence images, it is still not very well established. One of the main reasons for this could be that capturing intraoral photos is very time-consuming and technically demanding; however, this can be solved using intraoral scanners that can digitize the entire dentition within a short time [28,30].

In our study, the mean difference between whole-mouth biofilm-covered areas before and after rinsing with a 0.2% NaOCl solution was 38.99% for the percentual difference and 169.554 mm^2^ for the mean measured area, with SDs of 19.41 and 101.60, respectively. Comparison with data from the literature was difficult because the only existing study that conducted a full-mouth analysis of plaque reduction using the same measuring method was performed with tooth brushing as a plaque reduction method [29]. Earlier, another relatively similar study employed the same 3D scanning planimetric method, but included only the Ramfjord teeth for comparison, and, again, before and after teeth brushing [28]. The same team also compared the performance of two different brands of intraoral scan cameras for the evaluation of the reduction in dental plaque, again before and after tooth brushing [32]. However, in Guo’s et al. study, the comparison was made with respect to the clinical examination (Quigley-Hein Plaque Index of each tooth surface), while Jung et al. recorded a 43% decrease in comparison to the 3D scanning, very similar to our results. Nevertheless, the comparison between brushing and rinsing with dissolutive solutions may be inaccurate with regard to plaque reduction.

Furthermore, in our study, a detailed analysis of upper and lower arches performed. For the maxilla, the mean percentage of biofilm-covered area decreased from 475.85 mm^2^ to 294.53 mm^2^, resulting in a stained area variation of 181.32 mm^2^ (39.65%), while for the mandible, the mean percentage of biofilm-covered area decreased from 418.58 mm^2^ to 260.80 mm^2^, resulting in a stained area variation of 157.78 mm^2^ (38.26%). No comparison with data from the literature was available. Moreover, the plaque-covered area reduction seems not to be influenced by the location (maxilla or mandible), with a slightly bigger reduction in the maxilla.

This study has several limitations including its small sample size and short duration of the antimicrobial presence in the oral cavity. Our plaque analysis only relates to the surface area and not to the biofilm thickness. This not means that there is no correlation with the volume/thickness of the plaque, but also that—due to the convexity of the tooth surface—the measurements of approximal surfaces might be underestimated when compared to the buccal and lingual surfaces. The effect of the antimicrobial agent was limited by the duration of the exposure, which was dictated by the tolerability of the patients to the particular taste and smell of the rinsing solution.

The reduction in plaque-covered area, analyzed using the staining method and a 3D scanner, was less than empirically expected, also with regard to similar studies performed using brushing for plaque removal [28,29] and to the common knowledge about the high dissolutive properties of NaOCl, although the literature mentions 15% as a threshold for clinical efficiency [35]. However, it is worth mentioning that a NaOCl rinsing solution that could be more efficient in disrupting the oral biofilm is essentially one that is more concentrated (e.g., above 0.2%), which may not be tolerated by the patient [53]. On the other hand, currently recommended concentrations of NaOCl solutions, deemed to completely destroy the endodontic biofilm in daily practice (and under strict rubber dam protection), could rise up to 5%, which makes them absolutely unapplicable for oral rinsing. The analysis of the images revealed in all patients not only changes in the plaque-covered areas, but also changes in the color intensity of the residual plaque-covered areas, e.g., from deep purple to intense pink, sometimes two-toned. This might be due to the wash-up of the colorant, but also to the “thinning” of the biofilm pellicle under the dissolutive action of the rinsing solution, as observed under identical lighting conditions. It would be worth measuring the reductions in the thickness/volume of the biofilm layer in future studies using advanced methods, as these reductions are critical for controlling gingival inflammation.

## 5. Conclusions

Three-dimensional intraoral scanning could be a valuable method for direct quantitative measurement of the area-related amount of oral plaque, including therapeutic settings employing antimicrobial adjunctives to periodontal therapy during steps 1, 2 and 4. The 0.2% NaOCl rinsing solution seems to be a clinically efficient disruptor/dissolvent of the oral biofilm, both when integrated in modern protocols of periodontal therapy and for home self-care, resulting in relatively high reductions in plaque-covered areas.

## Figures and Tables

**Table 1:** Selected primary statistics for the upper arch. Area values before and after rinsing with NaOCl solution are given in mm^2^.

Upper Arch	Before Rinsing (mm^2^)	After Rinsing (mm^2^)	Difference (mm^2^)	Difference (%)
Minimum value	138.12 mm^2^	69.34 mm^2^	42.98 mm^2^	20.01 mm^2^ %
Maximum value	875.57 mm^2^	595.11 mm^2^	293.67 mm^2^	62.64 mm^2^ %
Range	737.45 mm^2^	525.76 mm^2^	250.69 mm^2^	42.63 mm^2^ %
Sum	7155.55 mm^2^	2356.26 mm^2^	1450.58 mm^2^	317.21 mm^2^ %
Mean	475.85 mm^2^	294.53 mm^2^	181.32 mm^2^	39.65 mm^2^ %
Median	512.54 mm^2^	240.19 mm^2^	174.00 mm^2^	40.74 mm^2^ %
Standard deviation	242.59 mm^2^	187.39 mm^2^	100.87 mm^2^	15.72 mm^2^ %
Variance	58,852.35 mm^2^	35,118.18 mm^2^	10,175.34 mm^2^	247.22 mm^2^ %

**Table 2:** Selected primary statistics for the lower arch. Area values before and after rinsing with NaOCl solution are given in mm^2^.

Lower Arch	Before Rinsing (mm^2^)	After Rinsing (mm^2^)	Difference (mm^2^)	Difference (%)
Minimum value	143.36 mm^2^	63.82 mm^2^	12.77 mm^2^	8.91 mm^2^ %
Maximum value	701.33 mm^2^	595.22 mm^2^	347.93 mm^2^	75.62 mm^2^ %
Range	557.96 mm^2^	531.39 mm^2^	335.16 mm^2^	66.71 mm^2^ %
Sum	3348.70 mm^2^	2086.42 mm^2^	1262.28 mm^2^	306.08 mm^2^ %
Mean	418.58 mm^2^	260.80 mm^2^	157.78 mm^2^	38.26 mm^2^ %
Median	383.98 mm^2^	270.09 mm^2^	149.81 mm^2^	42.86 mm^2^ %
Standard deviation	206.42 mm^2^	169.53 mm^2^	107.84 mm^2^	23.64 mm^2^ %
Variance	42,610.09 mm^2^	28,743.66 mm^2^	11,630.94 mm^2^	559.18 mm^2^ %

**Table 3:** Selected primary statistics cumulated for both arches (maxilla and mandible). Area values are given in mm^2^.

Full-Mouth Data	Before Rinsing (mm^2^)	After Rinsing (mm^2^)	Difference (mm^2^)	Difference (%)
Minimum value	138.12 mm^2^	63.82 mm^2^	12.77 mm^2^	8.91 mm^2^ %
Maximum value	875.57 mm^2^	595.22 mm^2^	347.93 mm^2^	75.62 mm^2^ %
Range	737.45 mm^2^	531.39 mm^2^	335.16 mm^2^	66.71 mm^2^ %
Sum	7155.55 mm^2^	4442.68 mm^2^	2712.86 mm^2^	623.29 mm^2^ %
Mean	447.22 mm^2^	277.66 mm^2^	169.55 mm^2^	38.95 mm^2^ %
Median	434.34 mm^2^	267.98 mm^2^	156.89 mm^2^	42.86 mm^2^ %
Standard deviation	219.59 mm^2^	173.50 mm^2^	101.60 mm^2^	19.41 mm^2^ %
Variance	48,223.69 mm^2^	30,105.57 mm^2^	10,324.01 mm^2^	376.83 mm^2^ %

**Table 4:** Basic statistical measures, basic confidence limits assuming normality, difference from the clinically significant value of 15% (Student’s *t*-test), and normality (Kolmogorov–Smirnov test) for the upper arches.

Basic Statistical Measures						
Location			Variability			
Mean	39.65		Std Deviation		15.72	
Median	40.74		Variance		247.23	
			Range		42.63	
			Interquartile Range		25.44	
Basic Confidence Limits Assuming Normality						
Parameter	Estimate		95% Confidence Limits			
Mean	39.65		26.51		52.80	
Tests for Location: Mu0 = 15						
Test	Statistic			*p* value		
Student’s *t*-test	t	4.43		Pr > ItI		0.003
Tests for Normality						
Test	Statistic			*p* value		
Kolmogorov–Smirnov		0.23		Pr > D		>0.150

**Table 5:** Basic statistical measures, basic confidence limits assuming normality, difference from the clinically significant value of 15% (student’s *t* test), and normality (Kolmogorov–Smirnov test) for the lower arches.

Basic Statistical Measures						
Location			Variability			
Mean	38.26		Std Deviation		23.65	
Median	42.86		Variance		559.18	
			Range		66.71	
			Interquartile Range		39.02	
Basic Confidence Limits Assuming Normality						
Parameter	Estimate		95% Confidence Limits			
Mean	38.26		18.49		58.03	
Tests for Location: Mu0 = 15						
Test	Statistic			*p* value		
Student’s *t*-test	t	2.78		Pr > ItI		0.027
Tests for Normality						
Test	Statistic			*p* value		
Kolmogorov–Smirnov	D	0.21		Pr > D		>0.150

## Data Availability

The data presented in this study are available on request from the corresponding author due to (privacy).
